# The Safe Use of a PTEN Inhibitor for the Activation of Dormant Mouse Primordial Follicles and Generation of Fertilizable Eggs

**DOI:** 10.1371/journal.pone.0039034

**Published:** 2012-06-27

**Authors:** Deepak Adhikari, Nagaraju Gorre, Sanjiv Risal, Zhiyi Zhao, Hua Zhang, Yan Shen, Kui Liu

**Affiliations:** Department of Chemistry and Molecular Biology, University of Gothenburg, Gothenburg, Sweden; University of Kansas Medical Center, United States of America

## Abstract

**Background:**

Primordial ovarian follicles, which are often present in the ovaries of premature ovarian failure (POF) patients or are cryopreserved from the ovaries of young cancer patients who are undergoing gonadotoxic anticancer therapies, cannot be used to generate mature oocytes for *in vitro* fertilization (IVF). There has been very little success in triggering growth of primordial follicles to obtain fertilizable oocytes due to the poor understanding of the biology of primordial follicle activation.

**Methodology/Principal Findings:**

We have recently reported that PTEN (phosphatase and tensin homolog deleted on chromosome ten) prevents primordial follicle activation in mice, and deletion of *Pten* from the oocytes of primordial follicles leads to follicular activation. Consequently, the PTEN inhibitor has been successfully used *in vitro* to activate primordial follicles in both mouse and human ovaries. These results suggest that PTEN inhibitors could be used in ovarian culture medium to trigger the activation of primordial follicle. To study the safety and efficacy of the use of such inhibitors, we activated primordial follicles from neonatal mouse ovaries by transient treatment with a PTEN inhibitor bpV(HOpic). These ovaries were then transplanted under the kidney capsules of recipient mice to generate mature oocytes. The mature oocytes were fertilized *in vitro* and progeny mice were obtained after embryo transfer.

**Results and Conclusions:**

Long-term monitoring up to the second generation of progeny mice showed that the mice were reproductively active and were free from any overt signs or symptoms of chronic illnesses. Our results indicate that the use of PTEN inhibitors could be a safe and effective way of generating mature human oocytes for use in novel IVF techniques.

## Introduction

In the mammalian ovary, the original pool of primordial follicles is the source of all eggs that will be produced over the entire course of the organism’s reproductive life. To maintain the normal length of the female’s reproductive life, the majority of primordial follicles must remain in a quiescent state for later use [1]–[4]. A highly controlled, but poorly understood, mechanism ensures that only a limited number of primordial follicles are activated at any given time to provide a steady supply of fertilizable oocytes that are available at regular intervals. However, during the pathological conditions such as premature ovarian failure (POF), there is an accelerated depletion of primordial follicles [5], [6]. Recently, the number of POF patients has increased dramatically due to the increasing number of survivors of childhood and adolescent cancers whose primordial follicles have been destroyed by toxic anticancer therapies [7].

Primordial follicles are located in the cortical region of the ovaries and are the most abundant type of follicles at any stage of the female’s life [8]. Because primordial follicles are resistant to freezing and thawing processes, cryopreservation of ovarian cortical tissue prior to gonadotoxic therapies has become an attractive fertility preservation technique [9]. Moreover, ovarian tissue cryopreservation remains the only fertility-preserving option for children because neither ovarian stimulation and collection of mature oocytes nor collection of fertilized embryos is feasible [7]. Thus, over the past decade, an increasing number of fertility centers have been cryopreserving ovarian tissues prior to gonadotoxic therapies, and much of this increase is coming from prepubescent patients [10]. The ovaries of adult POF patients may still contain certain numbers of primordial follicles, but these small follicles do not express the receptor for follicle stimulating hormone (FSH). Thus these follicles cannot be used with current *in vitro* fertilization (IVF) techniques in which FSH stimulation of the follicles is the first step in obtaining fertilizable eggs [2]. Nevertheless, ovarian cortical tissue from these women can still be collected without hyperstimulation and without regard to their menstrual cycle stage. Theoretically, it is possible to use these primordial follicles for the purpose of *in vitro* maturation (IVM) to obtain the mature, fertilization-competent oocytes that are required to restore fertility to these patients [3], [11].

Despite the huge potential of primordial follicle cultures to produce fertilizable oocytes *in vitro*, this technique has not been successful due to the poor understanding of the growth regulation of these follicles. Recent work from our lab has revealed that phosphatase and tensin homolog deleted on chromosome ten (PTEN), a negative regulator of phosphatidylinositol 3 kinase (PI3K), functions in oocytes to specifically suppress the primordial follicle activation [12], [13]. This finding clearly indicated the possibility of treating ovarian tissues with PTEN inhibitors to activate primordial follicles and allow them to grow to a stage where the follicles can respond to FSH.

Accordingly, PTEN inhibitor has been shown to effectively activate primordial follicles both in neonatal mouse ovaries and in human ovarian cortical tissues. These activated follicles subsequently developed into mature follicles and generated fertilizable oocytes after transplantation into the kidney capsules of ovariectomized recipient mice [14]. Although live mice had been obtained upon fertilization and embryo transfer, long term follow up on the fertility and general health status of the offspring was not performed.

Stringent testing of the safety of PTEN inhibitors must be carried out before they can be used for primordial follicle culture in humans. With the aim of testing the safety of PTEN inhibitors for the generation of fertilizable eggs, we activated primordial follicles in neonatal mouse ovaries by treatment with bpV(HOpic) and then transplanted the ovaries under the kidney capsules of recipient mice to obtain mature oocytes. Mature oocytes were fertilized *in vitro* and, upon embryo transfer, healthy, fertile progeny mice were obtained. The fertility of second-generation progeny mice also appeared to be unaffected and these mice had no obvious health issues. Moreover, despite PTEN’s known roles in tumor suppression [15], [16] and metabolic regulation [17] in various tissues, the mice obtained from this novel form of IVF did not show any overt signs or symptoms of chronic illnesses over a prolonged testing period. Thus, the use of PTEN inhibitors increases the yield of mature mouse eggs that can be fertilized to generate healthy offspring, and our results show that PTEN inhibitors have significant clinical potential for generating healthy and fertilizable human oocytes.

## Results

### Enhanced Follicular Growth by Transient PTEN Inhibition

**Figure 1:**
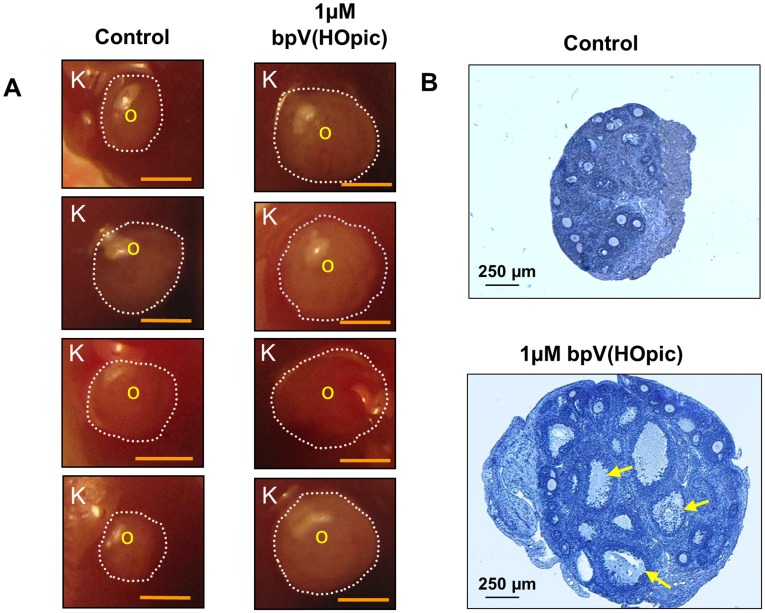
Enhanced follicular development by transient treatment of neonatal mouse ovaries with the PTEN inhibitor bpV(HOpic). (**A**) Comparison between the sizes of treated and control ovaries transplanted under the kidney capsules. One ovary from a PD3 mouse was cultured for 24 h with 1 µM bpV(HOpic) and another ovary was cultured without bpV(HOpic) and then transplanted under the capsule of each kidney of the same ovariectomized recipient as described in *Materials and Methods*. Ovaries that were treated with bpV(HOpic) before transplantation grew bigger than the non-treated control ovaries. K represents kidney tissue from the recipient, O represents the transplanted ovary, and the ovarian border is outlined by dashed circles. Scale bar = 1 mm. (**B**) Morphological analysis of treated and control ovaries excised from the kidney capsules. Ovaries from PD3 mice were cultured for 24 h with or without 1 µM bpV(HOpic) before transplantation under each kidney capsule of the same ovariectomized recipient as described in *Materials and Methods*. One day after the transplantation, recipient mice were treated daily with 2 IU of pregnant mare serum gonadotropin for 18 days. Fourteen hours before being killed, the mice were treated with 5 IU of human chorionic gonadotropin. Ovaries were excised from the kidney capsules and embedded in paraffin, and serial sections of 8 µm thickness were prepared and stained with hematoxylin. A larger number of antral follicles were observed in the bpV(HOpic)-treated ovaries (arrows) than in the control ovaries. The experiments were repeated at least 4 times, and 5 mice were used each time. Scale bar = 250 µm.

To determine the effect of transient PTEN inhibition on primordial follicle activation and subsequent follicular development, we removed both ovaries from postnatal day (PD) 3 mice. One of the ovaries was treated with bpV(HOpic) for 24 h and then transplanted under one side of the kidney capsules of an ovariectomized adult recipient mouse. As a control, the other ovary was cultured without bpV(HOpic) and was transplanted under the other kidney capsule in the same recipient mouse. We found that the bpV(HOpic)-treated ovaries were significantly larger than the untreated control ovaries (Fig. 1A). Moreover, ovarian histology showed that the treated ovaries had more follicles at preovulatory stages than untreated control ovaries (Fig. 1B).

### *In Vitro* Fertilization and Embryo Transfer to Obtain Live Pups

**Figure 2:**
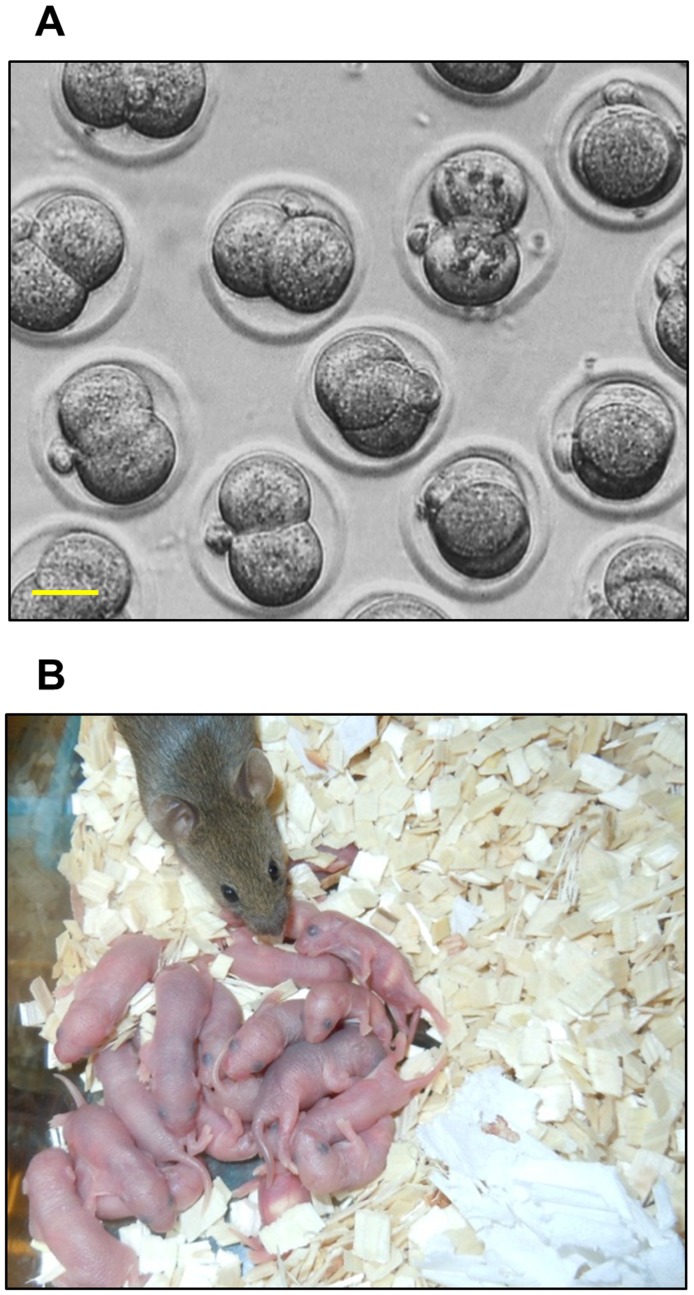
*In vitro* fertilization and birth of live pups after embryo transfer into the recipient mice. (**A**) Fertilization of mature oocytes obtained from the PTEN inhibitor-treated ovaries. Ovaries from PD3 mice were cultured for 24 h with 1 µM bpV(HOpic) before transplantation under the kidney capsules of ovariectomized recipient mice as described in the *Materials and Methods*. One day after the transplantation, recipients were treated daily with 2 IU of pregnant mare serum gonadotropin for 18 days. After 18 days, the recipient mice were injected with 5IU human chorionic gonadotropin and the grafted ovaries were collected 14 h later. MII stage oocytes were fertilized *in vitro* as described in the *Materials and Methods*. After 24 h post fertilization, embryos had reached the two-cell stage. Scale bar = 25 µM. (**B**) Birth of live pups after embryo transfer. *In vitro* fertilized two-cell embryos were transferred into the oviducts of pseudopregnant surrogate mothers that had been prepared as described in the *Materials and Methods*.

We isolated mature oocytes from the bpV(HOpic)-treated ovaries that had been transplanted under the kidney capsules of recipient mice. After 24 h of *in vitro* fertilization with donor sperm, the oocytes developed into two-cell stage embryos (Fig. 2A). A total of 149 two-cell embryos were transferred to the oviducts of 10 pseudopregnant recipient mice. We obtained 29 healthy, first generation (F1) pups (Fig. 2B) that grew into healthy adults. These results show that the use of a PTEN inhibitor for activation of primordial follicles does not affect the quality of the resulting eggs because the eggs can be fertilized and develop into adult mice.

### Effects of Treatment with bpV(HOpic) on the Fertility and Health of the First and Second Generation Progeny Mice

**Figure 3:**
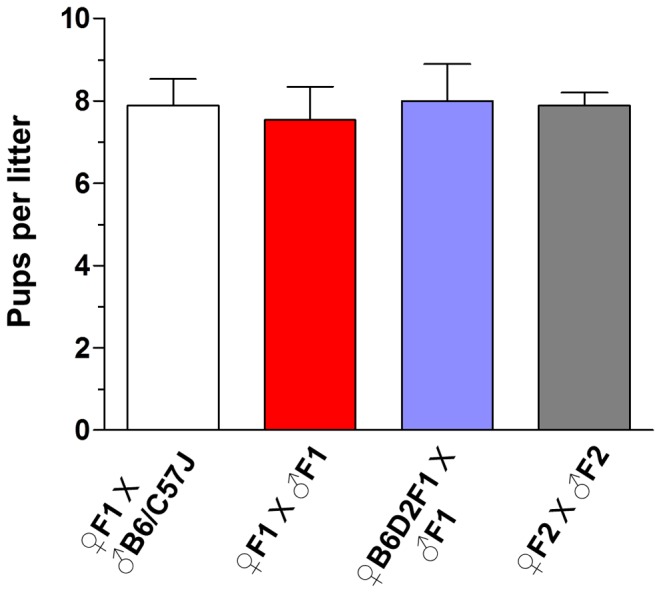
Fertility measurement of the first and second generation progeny mice. The F1 female and F1 male mice that were obtained by embryonic transfer were bred with B6/C57J male and B6D2F1 female mice, respectively. Fertility was also checked by breeding F1 males and F1 females. During the testing period, the mice regularly produced normal-sized F2 generation litters at normal intervals. To determine the fertility of the second generation mice, F2 females were bred with F2 males. n =  number of breeding pairs used.

To assess the possible impairment on the reproductive capacity of the progeny mice derived from bpV(HOpic)-treated oocytes, F1 males and F1 females were bred with either wild type (B6/C57J or B6D2F1 as indicated in the figure legends) mice or with their F1 siblings (Fig. 3). During an observation period lasting from 13 to 40 weeks of age, breeding between F1 females and B6/C57J males regularly produced second generation (F2) litters with an average litter size of 7.8±3.3 pups. Similarly, during the same testing period, breeding between B6D2F1 females and the F1 males regularly produced F2 generation litters with an average litter size of 8.0±3.1 pups (Fig. 3). To determine the fertility of breeding pairs when both parents were derived from the oocytes exposed to bpV(HOpic), we also bred F1 males with F1 females. We found that during the testing period breeding between F1 males and F1 females regularly generated F2 litters with an average litter size of 7.5±2.9 pups (Fig. 3). Thus, the F1 mice born as a result of IVF with oocytes treated with the PTEN inhibitor bpV(HOpic) were reproductively sound.

We next assessed the fertility of the second generation mice by recording the number of pups per litter produced by breeding between F2 females and F2 males. These F2 mice were obtained from the breeding between F1 females and B6/C57J males. Throughout an observation period lasting from 10 to 24 weeks of age, breeding between F2 females and F2 males produced third generation (F3) pups with an average litter size of 7.8±1.3 mice (Fig. 3). These results show that the transient treatment with bpV(HOpic), at a dose that is sufficient to trigger primordial follicle activation in neonatal mouse ovaries, does not impair the fecundity of the F1 and the F2 progenies.

Moreover, despite the known roles of PTEN as a tumor suppressor [15], [16] and a metabolic regulator [17], both the F1 (the first batch of these mice have been obtained over a year ago) and the F2 mice did not develop any tumors or other signs or symptoms of chronic illnesses.

### Fertility and Overall Health Status of Female Mice Injected with bpV(HOpic)

**Figure 4:**
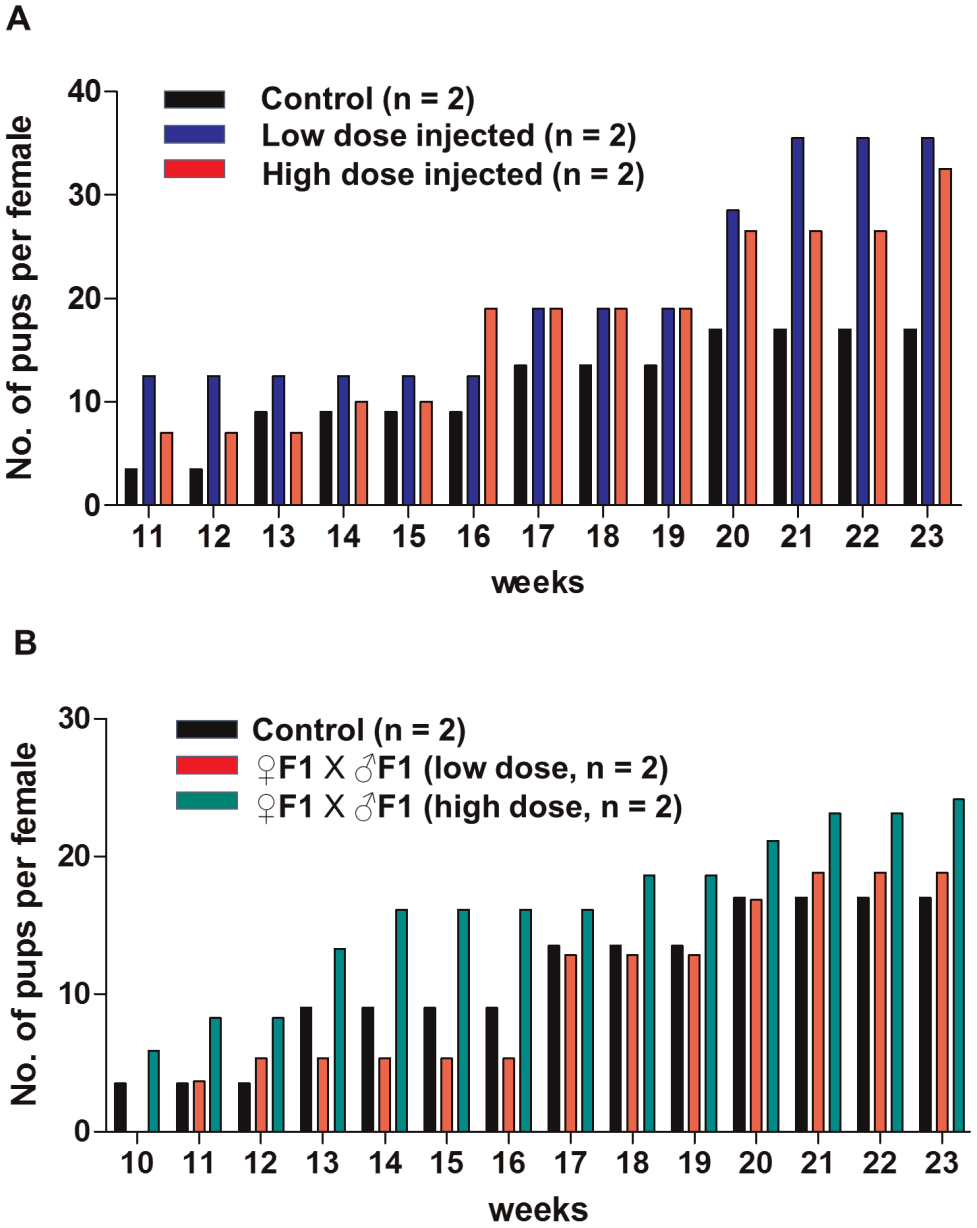
Fertility measurement of the female mice directly injected with bpV(HOpic). (**A**) Weekly comparison of the cumulative number of pups for the low dose bpV(HOpic)-injected mice (n = 2, blue bars), high dose-injected mice (n = 2, red bars) and control mice (n = 2, black bars). All mice had been bred with CD-1 strain males. (**B**) Weekly comparison of the cumulative number of pups produced by breeding the F1 mice. Breeding between F1 males and F1 females produced by low dose-injected females (n = 2, red bars), breeding between F1 males and F1 females produced by high dose-injected females (n = 2, green bars) and breeding between F1 males and F1 females produced by PBS-injected females (n = 2, black bars). n = number of breeding pairs used.

To gain more insight into the possible toxicity of bpV(HOpic), PD5 female mice of strain CD-1 were directly injected with this inhibitor. Injection was repeated again at PD18. One group of female mice was injected with a low dose (150 ng/g body weight) and the other group with a high dose (300 ng/g body weight) of bpV(HOpic). During a testing period from 10 to 23 weeks of age, both the low and high dose PTEN-inhibitor injected females regularly produced F1 litters with slightly higher numbers of pups than the control mice injected only with PBS (Fig. 4A). These mice also failed to develop tumors or other signs of chronic illnesses.

We extended our fertility and health status monitoring to the F1 mice that were produced by mice injected with both low and high doses of bpV(HOpic). Throughout the testing period from 10 to 23 weeks of age, breeding between F1 males and F1 females produced healthy F2 generation litters that were comparable in size to those produced by the control breeding pairs (Fig. 4B). Moreover, all of the bpV(HOpic)-injected mice and their F1 progeny mice were healthy and did not show any overt signs of chronic illness.

## Discussion

The development of an *in vitro* system for producing fertilization-competent oocytes from primordial follicles would revolutionize the current methods of treating female infertility. However, there has been only limited success in culturing primordial follicles to obtain mature oocytes [18] due to our limited knowledge of the biology of folliculogenesis, especially of how primordial follicles are activated from their dormant state [3]. Treatment of mouse ovaries and human cortical tissues with the PTEN inhibitor led to activation of primordial follicles in both mice and humans, which, upon prolonged transplantation under the kidney capsule of recipient mice, yielded significantly higher numbers of fertilizable oocytes than untreated controls [14]. These results clearly showed that PTEN inhibitors could indeed be used to activate primordial follicles in mice and human ovaries.

PTEN is a tumor suppressor that is mutated in many human tumors [15]. Similarly, conditional deletions of *Pten* from primordial germ cells, the prostate, the pancreas, mammary epithelium, thyrocytes, the liver, or the smooth muscle cell lineage in mice have been reported to be associated with teratomas, prostatic cancers, pancreatic cancers, breast cancers, thyroid cancers, cholangiocellular carcinomas, and leiomyosarcomas, respectively [reviewed in reference 3]. PTEN is also emerging as a regulator of metabolic pathways [17]. These potential negative effects of PTEN inhibition warrant stringent testing for possible tumorigenesis and metabolic illnesses such as diabetes, hypertension, and cardiovascular disease in the mice that are generated by the fertilization of oocytes obtained from PTEN inhibitor-treated ovaries. However, a comprehensive analysis of the fertility and overall health of mice derived from this technique has not yet been reported.

In the current study, we have cultured neonatal mouse ovaries with the PTEN inhibitor bpV(HOpic) and shown that the use of this compound can stimulate primordial follicle activation and results in the production of a higher number of mature follicles than in controls. The mature oocytes can be fertilized, and live mice can be obtained upon embryo transfer. We also showed that the progeny male and female mice are reproductively sound and healthy.

Despite the known antitumorigenic effect of PTEN in several tissue types, neither the oocyte specific [12] nor the granulosa cell specific [19] deletion of *Pten* led to ovarian tumor formation. Moreover, short term treatment of human [14, and our unpublished data] or bovine (our unpublished data) ovarian tissues with PTEN inhibitor, followed by long term transplantation under the kidney capsules of severe combined immunodeficient (SCID) mice did not lead to any tumor formation in the graft or in the recipient mice. These results indicate that PTEN has cell and tissue-specific functions and in the ovary it specifically acts as a suppressor of primordial follicle activation. In this regard, we have also shown that the deletion of *Pten* from oocytes does not affect the development of already growing follicles [13].

Successful *in vitro* culture of follicles for the production of mature oocytes that are competent for fertilization would be a major step forward in the development of treatment options for adult women and young girls facing potentially gonadotoxic diseases or treatments. In prepubescent girls who suffer from POF, ovarian cryopreservation is the only fertility-preserving option because neither ovarian stimulation and collection of mature oocytes nor obtaining fertilized embryos is feasible [9]. Even for adult POF patients who may still have large numbers of primordial follicles; such *in vitro* culturing techniques from ovarian cortical tissue would avoid the issues of menstrual cycles and requirements for hyperstimulation by hormones. Using this technique, hundreds of primordial follicles can be cryopreserved in a single procedure that can be performed immediately without causing delays in cancer treatment [20]. More importantly, in contrast to fully developed oocytes, primordial follicles are less susceptible to cryo-damage and are thus more easily preserved [21].

Cryopreserved ovarian tissue has been successfully reimplanted orthotopically and heterotopically to enhance follicular growth and obtain mature follicles [22], [23]. However, for cancer-POF patients the potential risk of reintroduction of cancer cells has always remained a concern during reimplantation [7]. In this regard, our successful use of a PTEN inhibitor for follicular activation is a very promising step towards obtaining a large number of mature oocytes *in vitro*. In the current study, the inhibitor-treated ovaries were transplanted under the kidney capsules of recipient mice to provide the optimum *in vivo* conditions for the growing follicles. Human follicular development, however, is a lengthy process that requires several months *in vivo* for mature follicles to develop [2]. Therefore, for the translation of this technique into humans, it is important to develop a culturing method that can support the full maturation of the activated follicles *in vitro*.

In summary, we have shown that transient inhibition of PTEN is a novel way of triggering the activation of primordial follicles, which, under favorable growth conditions, can develop into mature and fertilizable oocytes. These oocytes can then be used to obtain fertile and healthy progeny mice. Thus, our findings have significant clinical implications for treating female infertility by generating healthy, mature oocytes for *in vitro* fertilization.

## Materials and Methods

### Mice

Mice were obtained from Charles River Laboratories and were housed under controlled environmental conditions with free access to water and food. Illumination was on between 0600 and 1800 h. Ovaries of PD3 female mice were used for *in vitro* culture with bpV(HOpic) and transplantation under the kidney capsules of adult female mice of the same strain. Animals were anesthetized by intraperitoneal injection with 2,2,2-tribromoethanol (0.4 mg/g body weight; Avertin, Sigma).

Ethics Statement: Experimental protocols were approved by the regional ethical committee of the University of Gothenburg, Sweden.

### *In Vitro* Culture of Neonatal Mouse Ovary

Paired ovaries from PD3 B6D2F1 female mice were excised and washed three times in M2 medium (Sigma) supplemented with 3 mg/mL BSA (Sigma). Ovaries were cultured on Millicell inserts (Millipore) in 24-well plates (Nunc, Denmark) with 300 µL of culture medium. The culture medium consisted of α-MEM (Life Technologies) supplemented with 3 mg/mL BSA, 50 U/mL penicillin (Invitrogen), 50 µg/mL streptomycin (Invitrogen) and 2 mM L-glutamine (Invitrogen). From a given donor, one ovary served as the control and the other was treated with 1 µM bpV(HOpic) (Calbiochem). Ovaries were cultured for 24 h at 37°C in a humidified atmosphere of 5% CO_2_.

### Ovary Transplantation

The recipient mice were anesthetized and kidneys exteriorized through a dorso-horizontal incision. A small hole was torn in the kidney capsule using a 28 G needle. A control ovary was inserted into one kidney capsule and the bpV(HOpic)-treated ovary was inserted into the other kidney capsule of the same recipient mouse. In order to increase endogenous gonadotropin levels, both ovaries of the recipient mice were removed by cauterization at the top of the uterine horns. Finally, the body wall incisions and skin were closed. One day after the transplantation, recipients were treated daily with 2 IU of pregnant mare serum gonadotropin (PMSG) (Sigma) for 18 days.

### Histological Analysis of Ovary

Transplanted ovaries were excised from the kidney capsules of recipient mice, fixed in 4% paraformaldehyde, dehydrated, and embedded in paraffin. The paraffin-embedded ovaries were serially sectioned at 8 µm thickness and stained with hematoxylin for morphological observation.

### IVF and Embryo Transfer

Eighteen days after transplantation, the recipient mice were injected with 5 IU human chorionic gonadotropin (hCG) (Sigma) and the grafted ovaries were collected 14 h later in M2 medium. Ovaries were then punctured to release the oocytes into M2 medium containing 0.1% hyaluronidase (Sigma). The maturation stages of the oocytes were assessed and MII stage oocytes were used for IVF. Sperm were collected from 14 to16-week-old B6D2F1 male mice in human tubal fluid (HTF) medium (Millipore) and incubated under mineral oil (Sigma) for 1 h at 37°C in a humidified atmosphere of 5% CO_2_. The MII stage oocytes were placed in 500 µL drops of HTF medium with the sperm (2.5 × 10^5^/mL) and incubated for 5 h at 37°C with 5% CO_2_. After fertilization, the embryos were washed in KSOM medium (Millipore) to remove the excess sperm and then cultured in KSOM medium drops under mineral oil for 24 h at 37°C in a humidified atmosphere of 5% CO_2_. Two-cell embryos were transferred to the oviducts of pseudopregnant foster mothers. Eight to 10-week-old B6/CBA (F1) female mice were mated with vasectomized males of the B6D2F1 strain to prepare the foster mother before the embryo transfer.

### PTEN Inhibitor Injection in Mice

Female CD-1 mice were intraperitoneally injected with bpV(HOpic) (150 or 300 ng/g body weight) at 5 and 18 days after birth. The fertility of the treated females was assessed by counting the numbers of pups produced after mating with males of the same strain. To check the fertility of the F1 mice, male and female siblings were mated and the numbers of pups were counted.
